# An integrative approach for efficient analysis of whole genome bisulfite sequencing data

**DOI:** 10.1186/1471-2164-16-S12-S14

**Published:** 2015-12-09

**Authors:** Jong-Hun Lee, Sung-Joon Park, Nakai Kenta

**Affiliations:** 1 Department of Computational Biology and Medical Sciences, Graduate school of Frontier Sciences, the University of Tokyo 108-8639, Japan; 2 Human Genome Center, the Institute of Medical Science, the University of Tokyo, Tokyo 108-8639, Japan

**Keywords:** DNA methylation, whole genome bisulfite sequencing, bisulfite-read mapper

## Abstract

**Background:**

Whole genome bisulfite sequencing (WGBS) is a high-throughput technique for profiling genome-wide DNA methylation at single nucleotide resolution. However, the applications of WGBS are limited by low accuracy resulting from bisulfite-induced damage on DNA fragments. Although many computer programs have been developed for accurate detecting, most of the programs have barely succeeded in improving either quantity or quality of the methylation results. To improve both, we attempted to develop a novel integration of most widely used bisulfite-read mappers: Bismark, BSMAP, and BS-seeker2.

**Results:**

A comprehensive analysis of the three mappers revealed that the mapping results of the mappers were mutually complementary under diverse read conditions. Therefore, we sought to integrate the characteristics of the mappers by scoring them to gain robustness against artifacts. As a result, the integration significantly increased detection accuracy compared with the individual mappers. In addition, the amount of detected cytosine was higher than that by Bismark. Furthermore, the integration successfully reduced the fluctuation of detection accuracy induced by read conditions. We applied the integration to real WGBS samples and succeeded in classifying the samples according to the originated tissues by both CpG and CpH methylation patterns.

**Conclusions:**

In this study, we improved both quality and quantity of methylation results from WGBS data by integrating the mapping results of three bisulfite-read mappers. Also, we succeeded in combining and comparing WGBS samples by reducing the effects of read heterogeneity on methylation detection. This study contributes to DNA methylation researches by improving efficiency of methylation detection from WGBS data and facilitating the comprehensive analysis of public WGBS data.

## Background

DNA methylation, defined as the addition of methyl group on 5-carbon in cytosine, is a widely spread epigenetic mark. The DNA methylation pattern can serve to identify cells and guides cell development and tissue maintenance [1]. For decades, researchers have focused on methylation at CpG sites (mCpG) and found that differentially methylated regions (DMRs) among various cells are involved to cell-specific functions, aging and deceases [2-5]. Recently, methylation at CpH (mCpH; where H can be A, C, or T) sites has been confirmed to be a key regulator of brain development and embryonic stem cell (ESC) differentiation [6-9]. Therefore, profiling both mCpG and mCpH in a genome scale is crucial for understanding of various biological processes.

To analyze the methylation modifications, high-throughput methods coupled with microarray and next-generation sequencing have been widely used. Bisulfite microarray is a specially designed genotyping microarray combined with bisulfite treatment. Although this method is a useful strategy for targeted DNA methylation analyses, it is not suitable for genome-scale studies due to low genome coverage; only 0.8% of CpGs and 0.02% of CpHs have been covered in the newest version [10]. Reduced representation bisulfite sequencing (RRBS) [11] utilizes enzyme bindings to "CCGG" sites to detect information-rich regions, whereas the enzyme reaction leads experimental bias and limits the detection of mCpH [12]. Alternatively, as the widely-accepted gold standard method, whole genome bisulfite sequencing (WGBS) can detect both mCpG and mCpH at single nucleotide resolution in a genome scale [12].

For efficiently detecting the methylated sites with WGBS data, many computer programs have been developed. In particular, Bismark [13], BSMAP [14], and BS-seeker2 [15] are the most widely used bisulfite-read mappers that employ different strategies; BSMAP is a wild-card type mapper that converts all cytosine bases (Cs) of a reference genome to a letter Y and then aligns sequenced Cs and thymine bases (Ts) to the Y [12] by using SOAP [16]. Bismark and BS-seeker2 are three-letter type mappers that convert all Cs to Ts in both sequenced reads and a reference genome. Bismark and BS-seeker2 employ Bowtie2 [17] in global and local alignment modes, respectively. It has been reported that wild-card type mappers are better in mapping rate (percentage of reads being aligned) but struggle with mapping accuracy (percentage of reads mapped at correct positions) [12]. Three-letter type mappers show exactly opposite tendency [12]. Therefore, the choice of bisulfite-read mapper is an important issue for not only specific studies that use costly WGBS data but also comprehensive large-scale analyses of public WGBS datasets.

**Figure 1:**
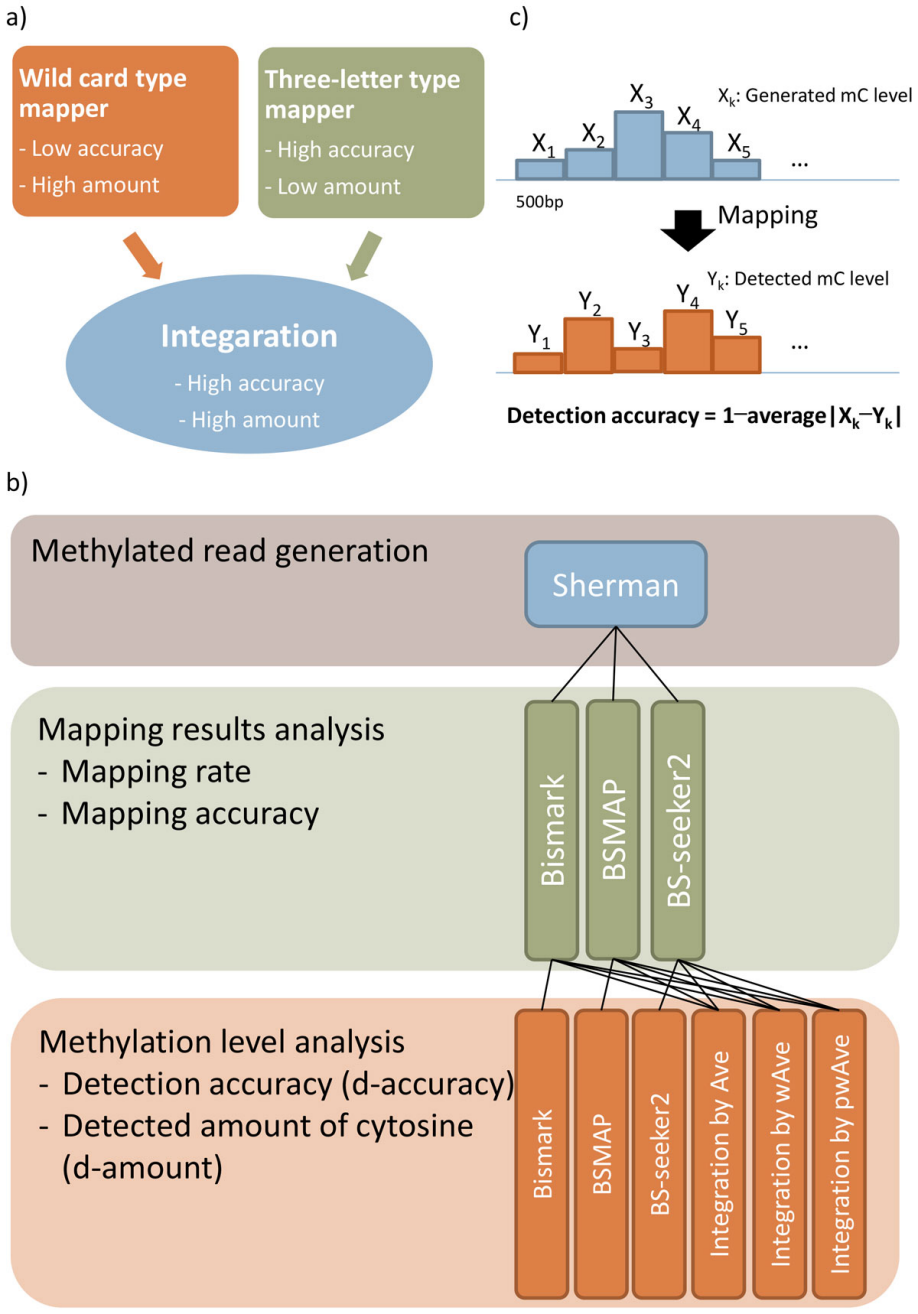
**Concept map and workflow of a research**. (a) Concept map for integrative approach. (b) Workflow of analysis and integration. We generated methylated reads by Sherman and mapped those reads with three mappers; Bismark, BSMAP, and BS-seeker2. Based on the analysis of the three mappers, we integrated the mapping results of the three mappers with three methods; Ave, wAve, and pwAve. Lastly, we evaluated the performances of the integrations with respect to detection accuracy (d-accuracy) and detected amount of cytosine (d-amount). (c) The d-accuracy was calculated by 1-difference between generated and detected methylation level at each block.

In this study, we investigated the performances of the three mappers on virtual WGBS dataset that has been simulated under various conditions. Through gathering detailed information, we confirmed that the mappers exhibit (dis)similar behaviors depending on the properties of simulated reads, which is consistent with results from previous studies [12,15]. Since the results showed that the behaviors of the three mappers were complementary to each other, we sought to integrate the characteristics of the mappers by scoring them to gain robustness against artifacts (e.g. sequencing errors and aligning errors). As a result, our integrative approach improved quality (i.e. the accuracy of the methylation detection at each C) and quantity (i.e. the number of detected Cs) of the methylation data with less dependency on the read properties (Figure 1a). We also applied our approach to public WGBS datasets of 13 tissues, and successfully grouped them according to their originated tissues by the patterns of mCpG and mCpH. We believe that this study contributes to DNA methylation researches by efficiently analyzing the WGBS data and facilitating comprehensive analyses of methylation patterns under the public WGBS data. In addition, this study gives a clue to algorithmic improvement of bisulfite-read mappers.

## Results and Discussion

### Overview of integrative approach

We integrated the methylation results from three bisulfite-read mappers: Bismark, BSMAP, and BS-seeker2. Bismark and BSMAP are the most widely used three-letter type and wild-card type mappers, respectively [18-20], and BS-seeker2 is the newest three-letter type mapper, which has shown higher mapping rate than Bismark [15].

The evaluation and integration of Bismark, BSMAP, and BS-seeker2 were conducted as described below (Figure 1b). First, bisulfite-read sets were generated by Sherman [21], with randomly designated methylation levels for every block of 500 base pairs (bps) in human chromosome 19. Then we mapped the reads by Bismark, BSMAP, and BS-seeker2 and evaluated the performances of the three mappers with respect to mapping rate and accuracy; the mapping rate is the portion of mapped read number over total read number, whereas the mapping accuracy is the portion of correctly mapped read number over mapped read number. Lastly, we integrated the methylation results from the mappers with three strategies and evaluated the performances in terms of detection accuracy (d-accuracy) and amount of detected Cs (d-amount). The d-accuracy was determined by the similarity between generated and detected methylation levels at each block (Figure 1c).

### Read-dependent performances of the three mappers

To investigate the performances of the three mappers under diverse read conditions, we analyzed the mapping results of the three mappers in the context of varying read quality, read length, and methylation levels.

**Figure 2:**
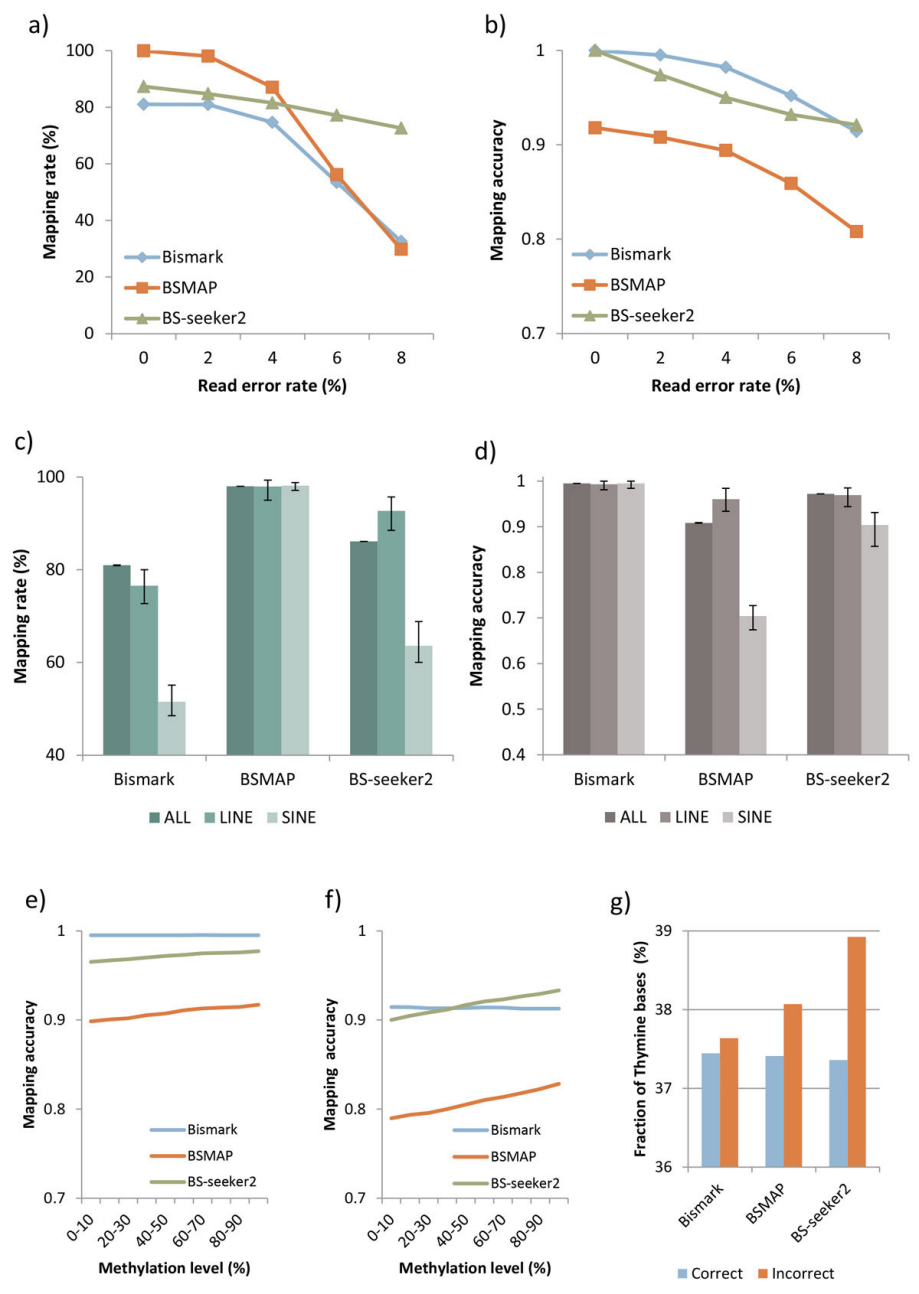
**Mapping results of the three mappers with short reads (length = 50 bps)**. (a) Mapping rate of the three mappers across read error rates. The mapping rate was calculated as mapped read number over total read number. (b) Mapping accuracy of the three mappers across read error rates. The mapping accuracy was calculated as the rate of correctly mapped read number over mapped read number. Mapping rate (c) and mapping accuracy (d) of the three mappers within all of hg19 chr19, LINEs, and SINEs. In this case, the read error rate was 2%. Mapping accuracy of the three mappers with reads in which error rate equals to 2% (e) and 8% (f). X-axis means methylation level in reads set at the bisulfite-read generation step. (g) Percentage of Ts in each read that were (in)correctly mapped by the three mappers.

For all three mappers, the mapping rate and mapping accuracy fluctuated with changes in read quality (Figure 2a, and 2b). When reads contained low error (<4%), BSMAP showed a higher mapping rate and lower mapping accuracy compared with others, consistent with previous studies [12,15]. As the read error rate increased (6-8%), the mapping rate of the BSMAP decreased dramatically, becoming lower than that of Bismark. Interestingly, for BS-seeker2, both mapping rate and mapping accuracy did not decreased substantially.

The read length also affected the performances of the three mappers (Additional file 1: Figure S1). We compared mapping results of 50 bp-long reads with those of 100 bp-long reads. When read error rate was low (2%), both mapping rate and mapping accuracy were higher within long reads. When read error rate was high (8%), mapping rate of Bismark and BSMAP were higher within short reads. Remarkably, the mapping accuracy of Bismark was also higher in short reads.

Additionally, we found that the performances of the three mappers varied greatly within repeat regions. In particular, the reads generated from short interspersed nuclear elements (SINEs) tended to be unmapped by Bismark and BS-seeker2 (Figure 2c) and incorrectly mapped by BSMAP (Figure 2d), which clearly showed the difference in performances between wild-card type and three-letter type mappers.

Lastly, we found that hypo-methylated reads tended to be incorrectly mapped by BSMAP and BS-seeker2 (Figure 2e, and 2f). This tendency was not found in the mapping results of Bismark. This may be explained in part by that the increased number of Ts, induced by the bisulfite conversion of unmethylated Cs, hindered the correct mapping of BSMAP and BS-seeker2. To confirm that, we measured the percentage of Ts in reads that correctly and incorrectly mapped by the three mappers. For BSMAP and BS-seeker2, the incorrectly mapped reads contained higher amount of Ts than the correctly mapped ones (Figure 2g).

In summary, Bismark, BSMAP, and BS-seeker2 performed differently in different read conditions. Bismark mapped reads with great accuracy and was not affected by the density of Ts in reads. However, Bismark tended to lose both mapping rate and accuracy when read error rate was higher in longer reads. BSMAP generally mapped a large number of reads to incorrect positions. Additionally, the mapping accuracy of BSMAP was affected by the density of Ts in reads. Both the mapping rate and mapping accuracy of BS-seeker2 were only slightly affected by the read error rate, whereas the mapping accuracy was affected by the density of Ts in reads.

### Integrative approach improved both the accuracy and amount of methylation detection

Based on the different performances of Bismark, BSMAP, and BS-seeker2 in varying read conditions, we integrated the mapping results of the three mappers using three strategies: Ave - average of the methylation levels from the three mappers, wAve - weighting by read depths, and pwAve - weighting by probabilistic method (i.e. Poisson distribution, see Method).

**Figure 3:**
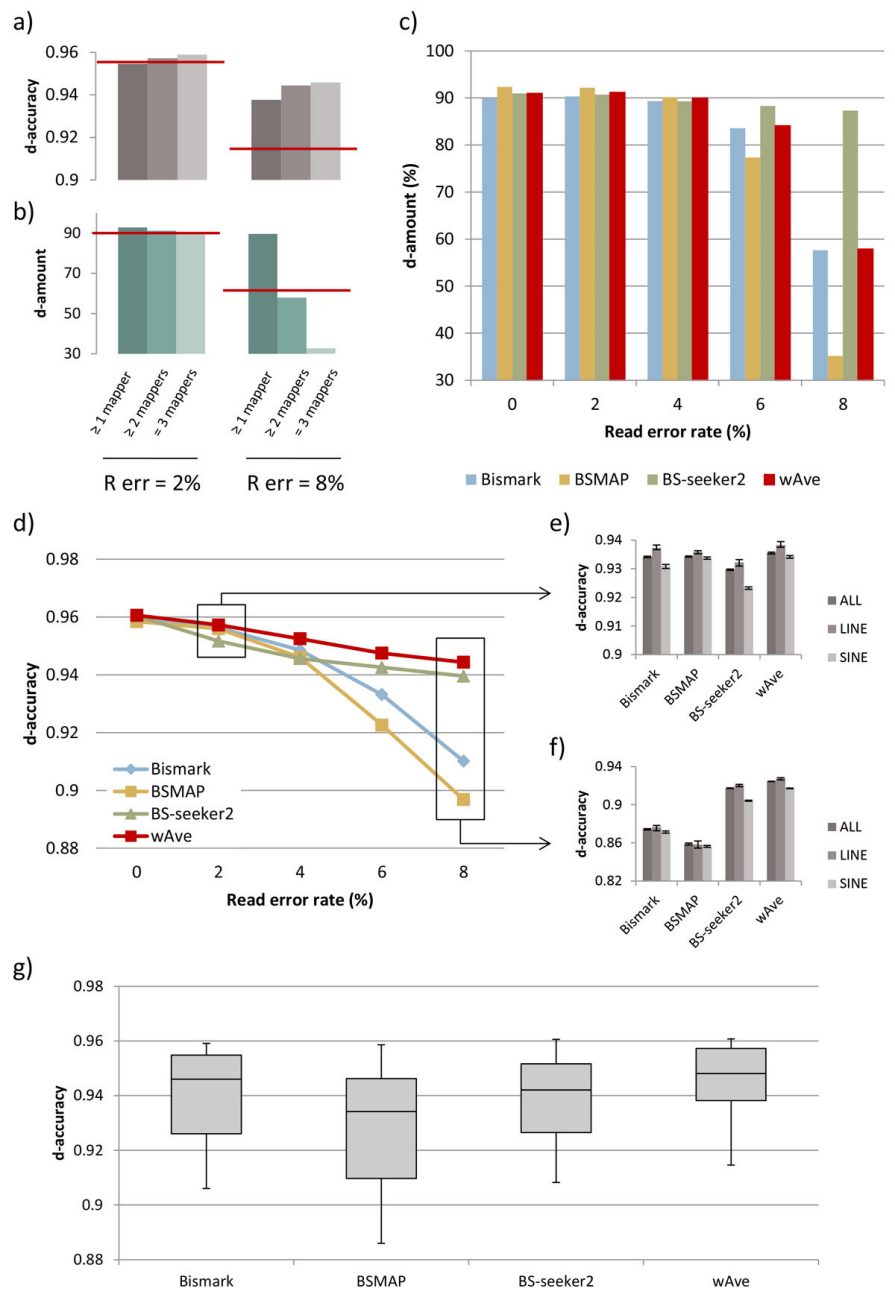
**Results with simulated reads (length = 100 bps)**. d-accuracy (a) and d-amount (b) of wAve when integrating positions covered by more than one, two and three mappers. R err means read error rate. The red lines represent average d-accuracy or d-amount of the three mappers. (c) Detected amount of CpGs by Bismark, BSMAP, BS-seeker2, and wAve. For wAve, the considered positions are those covered by more than two mappers. (d) d-accuracy of methylation levels at CpG sites across read error rates. The d-accuracy was 1-difference between generated and detected methylation level at each block. The d-accuracy within all of hg19 chr19, LINEs, and SINEs, in cases that read error rate equals to 2% (e) and 8% (f). The d-accuracies in repeat regions were determined by evaluating d-accuracy of each block, in which more than half is covered by repeat regions. (g) Distribution of d-accuracies within 100 read sets, in which read conditions were varied in length (50 bps and 100 bps) and error rates (from 0% to 8%).

We first examined the overlap of correctly mapped reads by the mappers. We found that 88.6% of high-quality 100 bps reads (2% read error rate) were correctly mapped by all the mappers, but this dramatically decreased to 6.7% in the case of low-quality reads (8% read error rate, Additional file 2: Figure S2). This result suggests the possibility that combining consensus of the mappers improves DNA methylation detection. Indeed, as the number of covering mappers (i.e. *n_i _*in Methods) increased, wAve improved the d-accuracy (Figure 3a). However, the d-amount was dramatically decreased, even becoming far lower than the average of the three mappers when *n_i _*=3 in the cases with high read error rate (Figure 3b). Taking account of this tradeoff, we choose *n_i _*≥2 that yields constantly higher d-amount than Bismark, and higher d-amount than BS-seeker2 or BSMAP in some cases (Figure 3c).

As shown in Table 1 among the three integration methods, wAve marked the highest d-accuracy in most read conditions, whereas pwAve showed the best d-accuracy in limited cases that short reads contain few errors (≤4%). The wAve remarkably improved d-accuracy compared with the individual mappers (Figure 3d). The Wilcoxon single-rank test over 500 bps blocks revealed significantly low P value (≤5.0E-2) in most of read conditions (Additional file 3: Tables S1-S4). Especially, the wAve increased d-accuracy within SINEs in which the mappers showed low mapping rate or accuracy (Figure 3e and 3f). Taken together, the wAve successfully improved the methylation detection compared with using individual mappers.

**Table 1:** d-accuracy of the integration methods

	Long reads (100 bps)					Short read (50 bps)				
**Read error (%)**	**0**	**2**	**4**	**6**	**8**	**0**	**2**	**4**	**6**	**8**

**Ave**	96.044	95.675	95.126	94.220	93.195	95.786	94.880	93.807	92.332	90.905

**wAve**	**96.056**	**95.724**	**95.242**	**94.748**	**94.434**	95.763	94.853	93.816	**92.553**	**91.506**

**pwAve**	96.050	95.691	95.146	94.324	93.430	**95.804**	**94.912**	**93.824**	92.386	91.024

* Numbers in bold are the highest value within each column

The superior performance of wAve may be explained by the correlation between d-accuracy and mapping rate. Using read depth as weight, the wAve considered mapping rate as a first element on determining the certainty of the methylation levels from each mapper. On the other hand, pwAve indirectly employed mapping accuracy on weighting by considering the characteristics of the mappers; a mapper that maps larger number of reads compared with other mappers tended to maps reads at incorrect positions. The tendency was clearly revealed within short reads containing low error, so the d-accuracy of pwAve was the highest among the integration methods in those read conditions. Generally, however, d-accuracy was more strongly correlated with mapping rate (Pearson correlation coefficient equals to 0.83) than mapping accuracy (Pearson correlation coefficient equals to 0.64, Additional file 4: Figure S3), resulting in the higher d-accuracy of wAve than that of pwAve in most read conditions.

It is noteworthy that the d-accuracy of wAve exhibited the reduced dependency on read conditions (i.e. read length and quality, Figure 3g). We checked the distribution of d-accuracies of the three mappers and wAve, gathered from mapping results of 100 read sets (see Method).The wAve shows relatively less variance of d-accuracies among varied read conditions. Especially, the wAve decreased the difference of d-accuracy between high and low read error cases (Figure 3d). This implies that the wAve successfully reduces the effects of heterogeneous read conditions on methylation detection, facilitating comprehensive analyses of methylation patterns among public WGBS samples from various experiments.

### The integrative approach facilitated the comprehensive analysis of public WGBS data

Next, we re-analyzed 13 WGBS samples that were generated from various experiments with different read length and quality. In particular, we included six brain samples and four pluripotent stem cells, in which significant amount of CpH methylation is accumulated [6-9].

**Figure 4:**
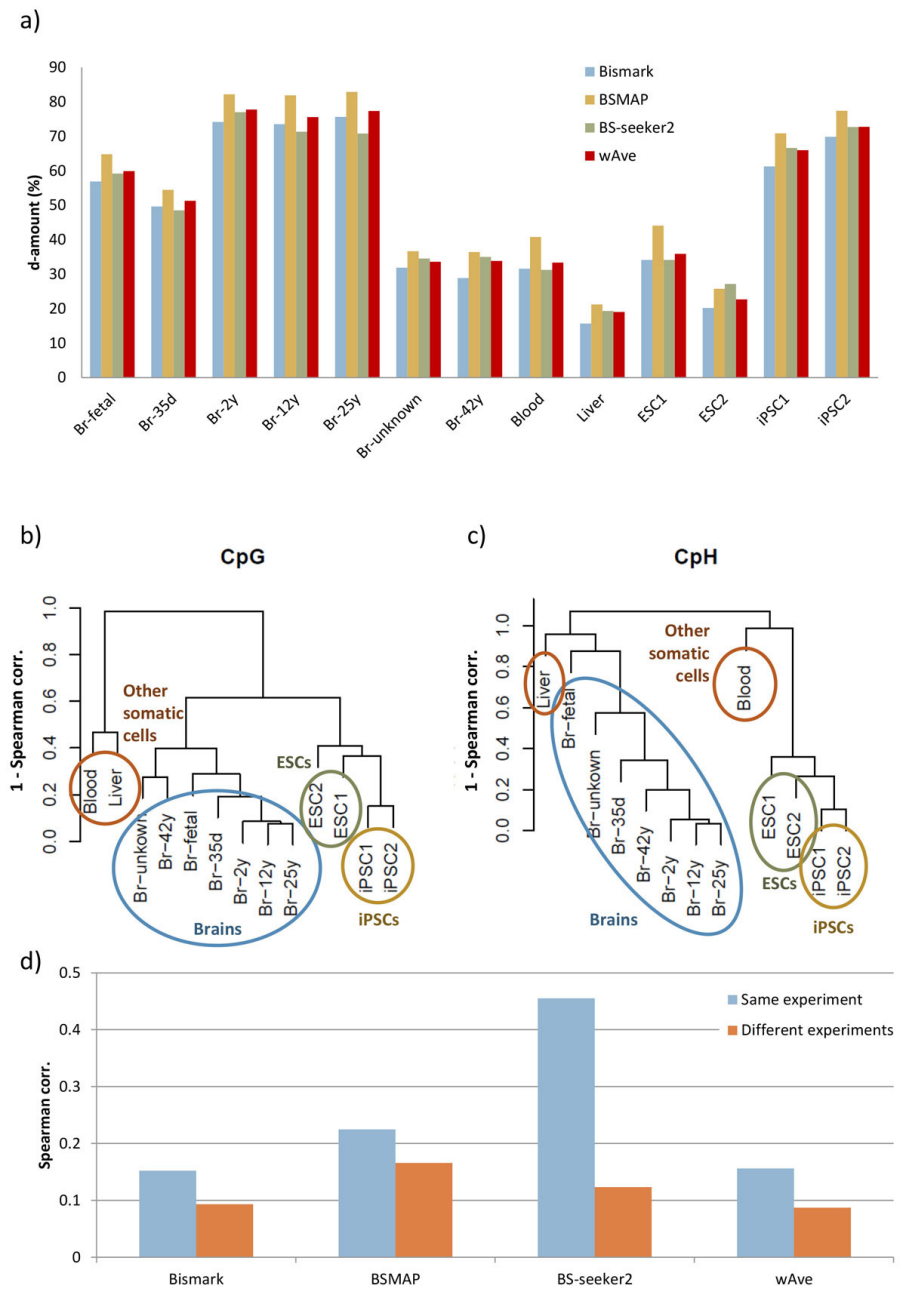
**Results with real WGBS data**. (a) d-amount of WGBS samples. ESC=Embryonic stem cell, iPSC = induced pluripotent stem cell, Br = brain, d = day and y = year (e.g. Br-5 y means 5 years old brain). Hierarchical clustering results based on CpG (b) and CpH (c) methylation levels of 10 kbps blocks across samples. The distance is 1-spearman correlation coefficient. (d) Spearman correlation coefficients of CpH methylation between liver with brain from same experiment (Br-unknown) and with a brain from different experiment (Br-42 y).

The mapping rate of WGBS samples was consistent with the mapping results of artificial bisulfite-reads (Additional file 5: Figure S4). BSMAP showed the highest mapping rate within nine samples, whereas BS-seeker2 showed the highest mapping rate within left four samples. Bismark showed the lowest mapping rate in most of the samples. In accordance with mapping rate, the d-amount by BSMAP was the highest among the three mappers within 12 samples. In contrast, BS-seeker2 showed the highest d-amount within only one sample. The d-amount by wAve was higher than Bismark within all samples (Figure 4a). Also, the wAve showed higher d-amount than BS-seeker2 within six samples.

We also examined the correlation between samples in terms of CpG and CpH methylation levels detected by the wAve. We found that both methylation levels clearly grouped samples according to their tissue of origin (Figure 4b and 4c). In particular, while brain samples were produced from three different experiments, they were closely positioned in the dendrogram. Moreover, an unknown-brain sample, a WGBS data from brain of which age was not known, and a sample from liver were produced from the same experiment but were successfully grouped according to each tissue type. These trends were not observed for BS-seeker2 (Additional file 6: Figure S5). The wAve clearly reduced false correlation between brain and liver from the same experiment (Figure 4d). We also found that the wAve results in significantly higher correlation of CpG methylation levels in gene-body regions within brain samples compared with that observed by Bismark (Additional file 7: Figure S6). Especially, the correlation among brain samples from different experiments were relatively more increased than that from same experiment. Thus, the wAve decreased the false correlation between samples from the same experiment and increased the correlation among samples from the same tissue.

## Conclusions

To efficiently detect DNA methylation from WGBS data, we analyzed and integrated the three most widely used bisulfite-read mappers, Bismark, BSMAP, and BS-seeker2. The procedure consisted of three steps: mapper analysis, analysis with simulated reads, and analysis with real WGBS dataset.

Firstly, we confirmed that with low read error rate, the performances of the three mappers were consistent with the results of former studies of wild-card type (e.g. BSMAP) and three-letter type (e.g. Bismark and BS-seeker2) [12]. In particular, the two types of mappers performed distinctly in SINEs, in which the wild-card type mappers falsely mapped reads, whereas the three-letter type mappers failed to map reads. It should be further investigated what distinction in algorithm induces the difference in mapping results in SINEs. In addition, the performances of Bismark and BSMAP dramatically decreased in case of high error reads, whereas BS-seeker2 did not affected much by the read error rates. Lastly, the mapping accuracies of BSMAP and BS-seeker2 were found to be dependent on the methylation level, whereas Bismark were not. Based on the complementary performances of the three mappers across varying read conditions, we integrated the mapping results of the three mappers with three methods: average (Ave), read depth-weighted average (wAve), and probabilistically weighted average by Poisson distribution (pwAve).

With the simulated reads, the wAve method resulted in significantly higher detection accuracy than that obtained with individual mappers and other integration methods. On the other hand, pwAve showed decreased accuracy compared with wAve. It should be further studied what probabilistic methods could improve the detection accuracy compared with read-depth weighting. In addition, the wAve exhibited higher detection of Cs than Bismark. Indeed, existing bisulfite mappers exhibit smaller increases in either quality or quantity of the methylation results compared with former systems. It is remarkable that the integration improved both the accuracy and amount of methylation detection. Furthermore, the integration reduced the dependency of detection accuracy on read conditions (i.e. error rate and length), proving that our method can facilitate the comprehensive analyses of multiple WGBS samples of which read conditions are heterogeneous.

With real WGBS samples, the wAve reduced the false correlation between WGBS samples generated from same experiments and increased the true correlation between those originated from same tissues. Thus, our method succeeded in facilitating comprehensive analyses of multiple WGBS datasets from various experiments by reducing the dependency of methylation results on read conditions.

In summary, our integrative approach improved both quality and quantity of methylation results from WGBS data, and facilitated the comprehensive analyses of DNA methylation among various read conditions. This study may contribute to researches about methylation patterns among samples in different conditions (e.g. tissue, age, or some diseases) by combining a massive public WGBS data. In addition, this study may give a new clue to algorithmic improvement of bisulfite-read mappers to enhance epigenetic researches.

## Methods

### Generating artificial sequenced reads

The sequenced reads were generated by Sherman [21]. The Sherman generates virtually bisulfite-treated reads with specific read number, length, error rate, and methylation level. We designated methylation level of CpG and CpH randomly and separately, on every 500 bps block of human genome (hg 19) chromosome 19. The human chromosome 19 is short but reveals the highest repeat rate among all chromosomes [22]. Therefore, we could effectively observe the diverse mapping results of the three bisulfite-read mappers with special focus on repeat regions. From the randomly methylated reference genome, we generated long (100 bps) and short (50 bps) reads separately. The numbers of generated reads were 50 for 100 bps reads and 100 for 50 bps reads in a block to adjust the average coverage depth equals to 10. Also, we generated reads with designating error rate to 0%, 2%, 4%, 6%, and 8%, in order to determine the dependency of mapping results on read error. We repeatedly generated all the cases of read sets 10 times. In total, we generated 100 read sets (2 read length cases × 5 read error cases × 10 repeat) for which the read number was 5.4 million and 10.8 million for long and short reads, respectively.

### Parameters for read generation

- For short reads

/Sherman -l 50 -n 100 -e [ERROR] --genome_folder [DIR] -CG [mCG] -CH [mCH]

- For long reads

/Sherman -l 100 -n 50 -e [ERROR] --genome_folder [DIR] -CG [mCG] -CH [mCH]

[DIR]: Directory to fasta file that include 500 bp-long sequences. Before running Sherman, human chromosome divided into 500 bps sequences and saved in separate directories.

[ERROR]: repeatedly set to 0, 2, 4, 6, and 8

[mCG] and [mCH]: randomly and independently set from 0 to 100. After the read generation completed, Sherman reported exact value of the bisulfite-treated rates on CpG and CpH positions. We used the reported values as designated methylation level at each block.

### Read mapping and extracting methylation level

We mapped both artificial reads and real WGBS reads with Bismark, BSMAP, and BS-seeker2. In mapping, we unified the maximum mismatch threshold to 4% of the read length, to determine the distinct performances of the three mappers in unified parameters. The command lines for each mapper were as below;

Bismark; we used bowtie2 as an aligner for better performance [13].

perl ./bismark -o [ouput] --bowtie2 [reference genome] [input fastq] --score_min L,0,-0.24

BSMAP; we set the maximum number of equal best hits to one [14].

./bsmap -a fastq file -d [reference genome] -o [output] -w 1 -v 0.04

BS-seeker2; we used bowtie2 as an aligner for better performance. [15]

python ./bs_seeker2-align.py -i [input fastq] -o [output] -g [reference genome] --aligner = bowtie2 -m 0.04

After mapping, we removed duplicates possibly induced by PCR amplification. The duplicated reads from Bismark, BSMAP, and BS-seeker2 were removed by picard [23], samtool rmdup [24], and a program of BS-seeker2 [15], respectively. After removing duplicates, methylation levels of each C were extracted by programs of each mapper. In results with simulated reads, we considered methylation levels at Cs that covered by more than one read. In results with real WGBS data, however, we considered methylation levels at Cs that covered by more than five reads in order to increase the confidence of methylation level at each C [25]. The methylation level at each C was calculated as the ratio of unconverted Cs over the total mapped read number.

### Integration of mapping results

The integration of three mappers was conducted at single base resolution. We extracted the number of both converted and un-converted Cs at each cytosine position. The methylation level *M_ij _*at the *i*th C position detected by a mapper *j *(=Bismark, BSMAP, BS-seeker2) was calculated as below;

$$M_{ij}=\frac{{N^{c}}_{ij}}{{N^{c}}_{ij}+{N^{t}}_{ij}},$$

where *N^c ^*and *N^t ^*are the number of Cs and Ts, respectively. If there is no mapped read by the mapper *j*, *M_ij _*is set to zero. We integrated *M_ij _*using three methods; *Ave *(average), *wAve *(weighted average) and *pwAve *(probabilistic weighted average). *Ave *was given by

$$Ave_{i}=\frac{\sum_{j}M_{ij}}{n_{i}},$$

where *n *is the number of mappers with constrain *M_ij _*> 0. *wAve *weights *M_ij _*by the read depth of mapper *j *with assuming that the methylation level detected by many reads is more confident. This is based on the observation that read-mapping rate and detection accuracy of methylation levels are correlated (Figure 2). The *wAve *was given by

$$wAve_{i}=\frac{\sum_{j}W_{ij}M_{ij}}{n_{i}},$$

$$W_{ij}=\frac{N_{ij}^{d}}{\sum_{j}N_{ij}^{d}},$$

where *W *and *N^d ^*is the weight and the read depth, respectively. *pwAve *uses Poisson distribution for weighting *M_ij_*. Based on the observations of the performances of the three mappers, we assumed that if a mapper mapped more reads than other mappers, the probability of existing incorrectly mapped reads at each position is also higher than that by other mappers. The *pwAve *was given by

$$pwAve_{i}=\frac{\sum_{j}W_{ij}^{p}M_{ij}}{n_{i}},$$

$$W_{ij}^{p}=\frac{f\left(N_{ij}^{d};\lambda_{j}\right)}{\sum_{j}f\left(N_{ij}^{d};\lambda_{j}\right)},$$

where *W^p ^*is the weight by the probability function *f *of Poisson distribution with parameter *λ *that is the average read depth of a mapper over whole genome.

### WGBS data preparation

We collected 13 WGBS samples from 5 experiments (Table 2). For evaluating the CpH methylation level, seven of human brains and four of pluripotent stem cells, known to have specific CpH methylation patterns [6,9,26], were included to the dataset. All the samples were quality-trimmed by fastx toolkit [27], with setting that minimum phred quality score equals to 20 and minimum read length equals to half of the original read length. We mapped all WGBS sample in single mode for the greatest generalization [20].

**Table 2:** WGBS data description

Tissue	Age	Read #	Length	Type	Experiment	Replica #
frontal cortex	fetal(20-week)	788M	100	Single	GSE47966[6]	9

middle frontal gyrus	35-day	669M	100	Single		12

middle frontal gyrus	2-year	900M	100	Single		12

middle frontal gyrus	12-year	594M	100	Single		12

middle frontal gyrus	25-year	903M	100	Single		12

prefrontal cortex	42-year	706M	100	Paired	GSE46710[28]	2

brain	Unknown	549M	90	Paired	GSE46698[29]	4

liver	Unknown	336M	100	Paired		7

ESC1		2655M	45	Single	GSE40832[30]	1

Blood		1240M	45	Single		1

ESC2		460M	87	Single	GSE16256[31]	5

iPSC1		667M	87	Single		13

iPSC2		837M	87	Single		20

- Parameters for quality-trimming

fastq_quality_trimmer -t 20 -l [half of read length] -i sample.fastq -o [output directory] -Q [phred score scale]

## Competing interests

The authors declare that they have no competing interests.

## Authors' contributions

JHL designed and conducted the data analyses. JHL and SJP wrote the manuscript. KN conceived and designed the study. All authors read and approved the final manuscript.

## Supplementary Material

Additional file 1 **Figure S1 - Mapping results of the three mappers with long (100 bp) and short (50 bp) reads**. The bars show mapping rate and mapping accuracy of the three mappers with reads that contains 2% error (a and c for mapping rate and mapping accuracy, respectively) and 8% error (b and d for mapping rate and mapping accuracy, respectively). The dark purple bars represent results with short (50 bp) reads and the light purple bars represent results with long (100 bp) reads. (Format: PDF) Click here for file

Additional file 2 **Figure S2 - Rate of correctly mapped reads by three mappers**. The numbers show rate of reads that correctly mapped by each three mapper over total read number, when read error rate equals to 2% (a) and 8% (b). The numbers in middle reveal rate of reads that correctly mapped by all three mappers. Also the numbers followed by each mapper shows rate of reads that correctly mapped only by the mapper. (Format: PDF) Click here for file

Additional file 3 **Tables S1-S4 - one-side Wilcoxon single-rank test between d-accuracy by wAve and d-accuracy by three mappers**. The tables show P-values by one-side Wilcoxon single-rank test between d-accuracy by wAve and d-accuracy by three mappers across 500-long blocks of hg19 chr19. If the P-value is lower than 0.05, it means the d-accuracy by wAve is significantly lower than the d-accuracy by each mapper across blocks. The bold types are values that lower than 0.05. R err means read error rate. (Format: PDF) Click here for file

Additional file 4 **Figure S3 - Correlation of detection accuracy with mapping accuracy and mapping read**. It shows proportional relationship between detection accuracy with mapping accuracy (a), and detection accuracy with mapping rate (b). Each point represents mapping results by Bismark, BSMAP and BS-seeker2 with read sets in which the read error rates equal to 0%, 2%, 4%, 6% and 8%, and read lengths equal to 50 bp and 100 bp. (Format: PDF) Click here for file

Additional file 5 **Figure S4 - Mapping rate of whole genome bisulfite sequencing data**. It shows mapping rates of WGBS samples by the three mappers; ESC = Embryonic stem cell, iPSC=induced pluripotent stem cell, Br = brain, d = day and y = year (cf. Br-5 y means 5 years old brain) (Format: PDF) Click here for file

Additional file 6 **Figure S5 - Hierarchical clustering results base on CpG and CpH methylation levels extracted by BS-seeker2**. Hierarchical clustering results base on CpG and CpH methylation levels extracted by BS-seeker2; Distance is 1-spearman correlation coefficient. ESC = Embryonic stem cell, iPSC = induced pluripotent stem cell, Br = brain, d = day and y = year (cf. Br-5 y means 5 years old brain). Also, the red circle groups the two samples that produced by same experiment. (Format: PDF) Click here for file

Additional file 7 **Figure S6 - Correlation of CpG methylation levels among brain samples that produced from same experiment and different experiments**. Spearman correlation of CpG methylation in gene-body regions between brain samples that produced from same experiment (GSE47966[6], 5 samples) and multiple experiments (GSE47966[6], GSE46710[28], GSE46698[29], 7 samples). Error bars represent maximum and minimum correlation value between samples. The information of gene-body regions was downloaded from refseq database. (Format: PDF) Click here for file

## Declaration

Publication charges for this article have been funded by JSPS KAKENHI Grant Number 25290067.

This article has been published as part of *BMC Genomics *Volume 16 Supplement 12, 2015: Joint 26th Genome Informatics Workshop and 14th International Conference on Bioinformatics: Genomics. The full contents of the supplement are available online at http://www.biomedcentral.com/bmcgenomics/supplements/16/S12.

## References

[B1] Meissner A Genome-scale DNA methylation maps of pluripotent and differentiated cells Nature 2008 454 7205 766 70 18600261 10.1038/nature07107 PMC2896277

[B2] Day K Differential DNA methylation with age displays both common and dynamic features across human tissues that are influenced by CpG landscape Genome Biol 2013 14 9 R102 24034465 10.1186/gb-2013-14-9-r102 PMC4053985

[B3] Varley KE Dynamic DNA methylation across diverse human cell lines and tissues Genome Res 2013 23 3 555 67 23325432 10.1101/gr.147942.112 PMC3589544

[B4] Horvath S DNA methylation age of human tissues and cell types Genome Biol 2013 14 10 R115 24138928 10.1186/gb-2013-14-10-r115 PMC4015143

[B5] Baylin SB DNA methylation and gene silencing in cancer Nat Clin Pract Oncol 2005 2 Suppl 1 S4 11 16341240 10.1038/ncponc0354

[B6] Lister R Global epigenomic reconfiguration during mammalian brain development Science 2013 341 6146 1237905 23828890 10.1126/science.1237905 PMC3785061

[B7] Ziller MJ Genomic distribution and inter-sample variation of non-CpG methylation across human cell types PLoS Genet 2011 7 12 e1002389 22174693 10.1371/journal.pgen.1002389 PMC3234221

[B8] Guo JU Distribution, recognition and regulation of non-CpG methylation in the adult mammalian brain Nat Neurosci 2014 17 2 215 22 24362762 10.1038/nn.3607 PMC3970219

[B9] Lister R Human DNA methylomes at base resolution show widespread epigenomic differences Nature 2009 462 7271 315 22 19829295 10.1038/nature08514 PMC2857523

[B10] Morris TJ S Beck Analysis pipelines and packages for Infinium HumanMethylation450 BeadChip (450k) data Methods 2015 72 3 8 25233806 10.1016/j.ymeth.2014.08.011 PMC4304832

[B11] Meissner A Reduced representation bisulfite sequencing for comparative high-resolution DNA methylation analysis Nucleic Acids Res 2005 33 18 5868 77 16224102 10.1093/nar/gki901 PMC1258174

[B12] Bock C Analysing and interpreting DNA methylation data Nat Rev Genet 2012 13 10 705 19 22986265 10.1038/nrg3273

[B13] Krueger F Andrews SR Bismark: a flexible aligner and methylation caller for Bisulfite-Seq applications Bioinformatics 2011 27 11 1571 2 21493656 10.1093/bioinformatics/btr167 PMC3102221

[B14] Xi Y Li W BSMAP: whole genome bisulfite sequence MAPping program BMC Bioinformatics 2009 10 232 19635165 10.1186/1471-2105-10-232 PMC2724425

[B15] Guo W BS-Seeker2: a versatile aligning pipeline for bisulfite sequencing data BMC Genomics 2013 14 774 24206606 10.1186/1471-2164-14-774 PMC3840619

[B16] Alper BS SOAP: solutions to often asked problems. Choice of antihistamines for urticaria Arch Fam Med 2000 9 8 748 51 10927716 10.1001/archfami.9.8.748

[B17] Langmead B Salzberg SL Fast gapped-read alignment with Bowtie 2 Nat Methods 2012 9 4 357 9 22388286 10.1038/nmeth.1923 PMC3322381

[B18] Chatterjee A Comparison of alignment software for genome-wide bisulphite sequence data Nucleic Acids Res 2012 40 10 e79 22344695 10.1093/nar/gks150 PMC3378906

[B19] Tran H Objective and comprehensive evaluation of bisulfite short read mapping tools Adv Bioinformatics 2014 2014 472045 24839440 10.1155/2014/472045 PMC4009243

[B20] Kunde-Ramamoorthy G Comparison and quantitative verification of mapping algorithms for whole-genome bisulfite sequencing Nucleic Acids Res 2014 42 6 e43 24391148 10.1093/nar/gkt1325 PMC3973287

[B21] Sherman - bisulfite-treated Read FastQ Simulator http://www.bioinformatics.babraham.ac.uk/projects/sherman/

[B22] Grover D Alu repeat analysis in the complete human genome: trends and variations with respect to genomic composition Bioinformatics 2004 20 6 813 7 14751968 10.1093/bioinformatics/bth005

[B23] Picard http://broadinstitute.github.io/picard/

[B24] Samtools rmdup http://samtools.sourceforge.net/

[B25] Ziller MJ Charting a dynamic DNA methylation landscape of the human genome Nature 2013 500 7463 477 81 23925113 10.1038/nature12433 PMC3821869

[B26] Guo W Characterizing the strand-specific distribution of non-CpG methylation in human pluripotent cells Nucleic Acids Res 2014 42 5 3009 16 24343027 10.1093/nar/gkt1306 PMC3950701

[B27] http://hannonlab.cshl.edu/fastx_toolkit/index.html

[B28] Wen L Whole-genome analysis of 5-hydroxymethylcytosine and 5-methylcytosine at base resolution in the human brain Genome Biol 2014 15 3 R49 24594098 10.1186/gb-2014-15-3-r49 PMC4053808

[B29] Court F Genome-wide parent-of-origin DNA methylation analysis reveals the intricacies of human imprinting and suggests a germline methylation-independent mechanism of establishment Genome Res 2014 24 4 554 69 24402520 10.1101/gr.164913.113 PMC3975056

[B30] Whole Genome Bisulfite Sequencing by ENCODE/HAIB http://www.ncbi.nlm.nih.gov/geo/query/acc.cgi?acc=GSE40832

[B31] UCSD Human Reference Epigenome Mapping Project http://www.roadmapepigenomics.org/

