# Supplementary material for: An integrative approach for efficient analysis of whole genome bisulfite sequencing data
Source: BMC Genomics. 2015 Dec 9;16(Suppl 12):S14. doi: 10.1186/1471-2164-16-S12-S14 (PMC4682396; doi:10.1186/1471-2164-16-S12-S14)

**Additional file 1: Figure S1 - Mapping results of the three mappers with long (100bp) and short (50bp) reads**

The bars show mapping rate and mapping accuracy of the three mappers with reads that contains 2% error (a and c for mapping rate and mapping accuracy, respectively) and 8% error (b and d for mapping rate and mapping accuracy, respectively). The dark purple bars represent results with short (50bp) reads and the light purple bars represent results with long (100bp) reads.

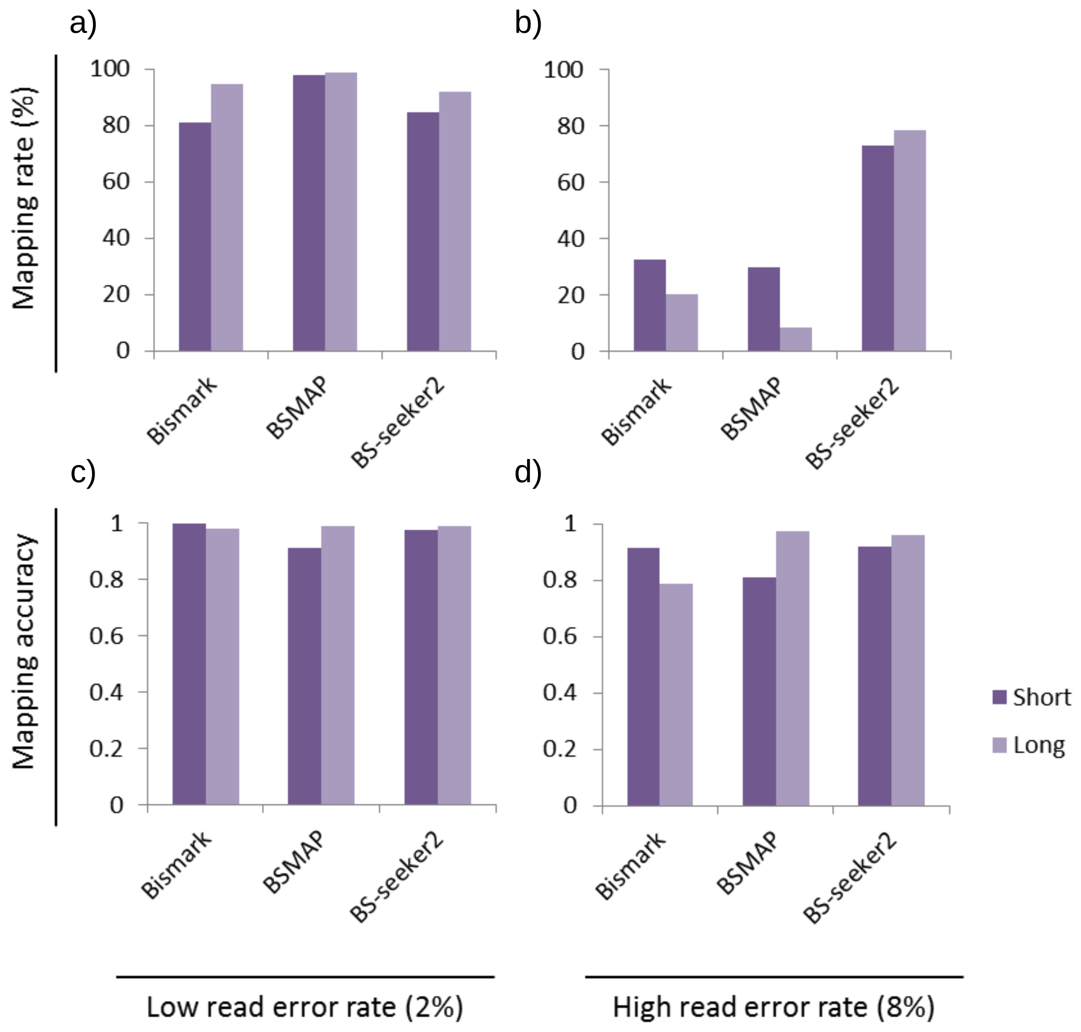

Supplement: Additional file 1 — Figure S1 - Mapping results of the three mappers with long (100 bp) and short (50 bp) reads. The bars show mapping rate and mapping accuracy of the three mappers with reads that contains 2% error (a and c for mapping rate and mapping accuracy, respectively) and 8% error (b and d for mapping rate and mapping accuracy, respectively). The dark purple bars represent results with short (50 bp) reads and the light purple bars represent results with long (100 bp) reads. (Format: PDF) [file 1471-2164-16-S12-S14-S1.pdf]
