# Supplementary material for: An integrative approach for efficient analysis of whole genome bisulfite sequencing data
Source: BMC Genomics. 2015 Dec 9;16(Suppl 12):S14. doi: 10.1186/1471-2164-16-S12-S14 (PMC4682396; doi:10.1186/1471-2164-16-S12-S14)

Additional file 2: Figure S2 - Rate of correctly mapped reads by three mappers

The numbers show rate of reads that correctly mapped by each three mapper over total read number, when read error rate equals to 2% (a) and 8% (b). The numbers in middle reveal rate of reads that correctly mapped by all three mappers. Also the numbers followed by each mapper shows rate of reads that correctly mapped only by the mapper.

a)

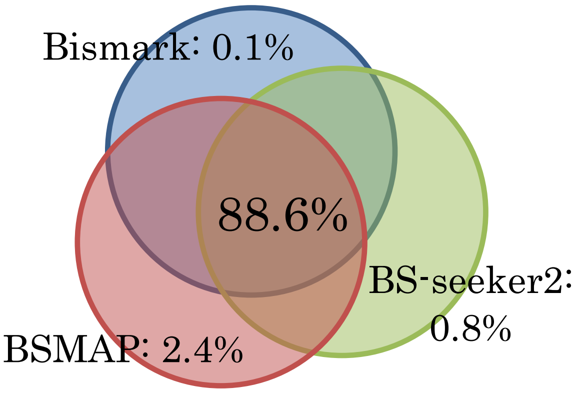

b)

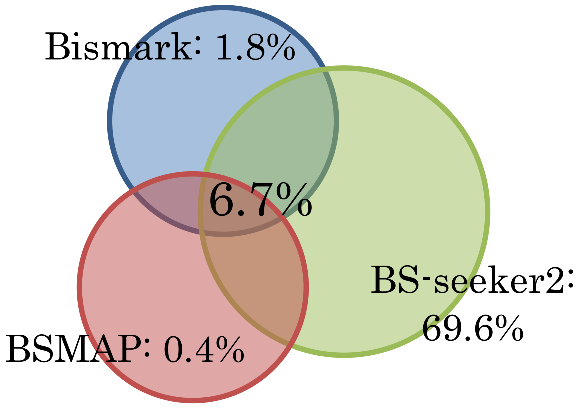

Supplement: Additional file 2 — Figure S2 - Rate of correctly mapped reads by three mappers. The numbers show rate of reads that correctly mapped by each three mapper over total read number, when read error rate equals to 2% (a) and 8% (b). The numbers in middle reveal rate of reads that correctly mapped by all three mappers. Also the numbers followed by each mapper shows rate of reads that correctly mapped only by the mapper. (Format: PDF) [file 1471-2164-16-S12-S14-S2.pdf]
