# Supplementary material for: An integrative approach for efficient analysis of whole genome bisulfite sequencing data
Source: BMC Genomics. 2015 Dec 9;16(Suppl 12):S14. doi: 10.1186/1471-2164-16-S12-S14 (PMC4682396; doi:10.1186/1471-2164-16-S12-S14)
Supplement: Additional file 3 — Tables S1-S4 - one-side Wilcoxon single-rank test between d-accuracy by wAve and d-accuracy by three mappers. The tables show P-values by one-side Wilcoxon single-rank test between d-accuracy by wAve and d-accuracy by three mappers across 500-long blocks of hg19 chr19. If the P-value is lower than 0.05, it means the d-accuracy by wAve is significantly lower than the d-accuracy by each mapper across blocks. The bold types are values that lower than 0.05. R err means read error rate. (Format: PDF) [file 1471-2164-16-S12-S14-S3.pdf]

Additional file 3: Table S1-4 one-side Wilcoxon single-rank test between d-accuracy by wAve and d-accuracy by three mappers

The tables show P-values by one-side Wilcoxon single-rank test between d-accuracy by wAve and d-accuracy by three mappers across 500-long blocks of hg19 chr19. If the P-value is lower than 0.05, it means the d-accuracy by wAve is significantly lower than the d-accuracy by each mapper across blocks. The bold types are values that lower than 0.05. R err means read error rate.

Table1 P-value for mCpG with 100bps-long reads

R err (%)	Bismark	BSMAP	BSseeker2
0	2.9E-21	5.3E-09	**3.7E-01**
2	2.3E-14	6.4E-03	4.0E-140
4	2.8E-96	5.0E-157	6.3E-103
6	0	0	8.7E-46
8	0	0	1.5E-62

Table2 P-value for mCpH with 100bps-long reads

R err (%)	Bismark	BSMAP	BSseeker2
0	7.7E-05	2.1E-04	**8.8E-01**
2	1.6E-07	8.6E-07	2.2E-57
4	5.9E-64	1.0E-152	1.9E-66
6	0	0	1.16E-28
8	0	0	1.12E-85

Table3 P-value for mCpG with 50bps-long reads

R err (%)	Bismark	BSMAP	BSseeker2
0	7.4E-63	8.0E-302	2.5E-07
2	**1.0E+00**	1.4E-184	5.9E-129
4	**1.0E+00**	1.3E-193	9.0E-296
6	1.99E-54	0	6.12E-152
8	0	0	2.36E-54

Table4 P-value for mCpH with 50bps-long reads

R err (%)	Bismark	BSMAP	BSseeker2
0	1.0E-38	8.6E-152	2.6E-06
2	**1.6E-01**	3.7E-131	9.2E-80
4	**9.6E-01**	3.1E-148	4.0E-150
6	7.75E-92	0	4.72E-87
8	0	0	4.08E-34
