# Supplementary material for: An integrative approach for efficient analysis of whole genome bisulfite sequencing data
Source: BMC Genomics. 2015 Dec 9;16(Suppl 12):S14. doi: 10.1186/1471-2164-16-S12-S14 (PMC4682396; doi:10.1186/1471-2164-16-S12-S14)

Additional file 4: Figure S3 – Correlation of detection accuracy with mapping accuracy and mapping read

It shows proportional relationship between detection accuracy with mapping accuracy (a), and detection accuracy with mapping rate (b). Each point represents mapping results by Bismark, BSMAP and BS-seeker2 with read sets in which the read error rates equal to 0%, 2%, 4%, 6% and 8%, and read lengths equal to 50bp and 100bp.

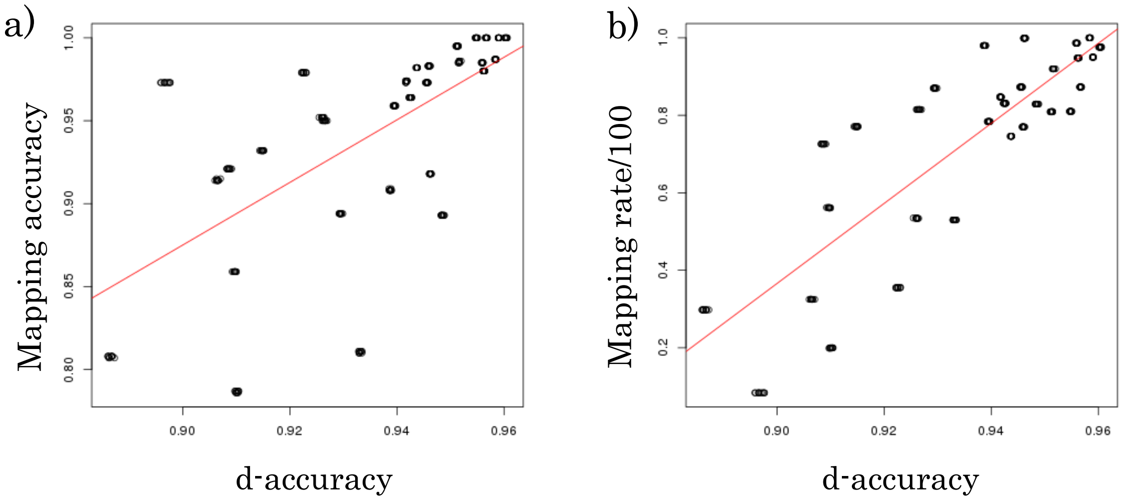

Supplement: Additional file 4 — Figure S3 - Correlation of detection accuracy with mapping accuracy and mapping read. It shows proportional relationship between detection accuracy with mapping accuracy (a), and detection accuracy with mapping rate (b). Each point represents mapping results by Bismark, BSMAP and BS-seeker2 with read sets in which the read error rates equal to 0%, 2%, 4%, 6% and 8%, and read lengths equal to 50 bp and 100 bp. (Format: PDF) [file 1471-2164-16-S12-S14-S4.pdf]
