# Supplementary material for: An integrative approach for efficient analysis of whole genome bisulfite sequencing data
Source: BMC Genomics. 2015 Dec 9;16(Suppl 12):S14. doi: 10.1186/1471-2164-16-S12-S14 (PMC4682396; doi:10.1186/1471-2164-16-S12-S14)

Additional file 5: Figure S4 – Mapping rate of whole genome bisulfite sequencing data

It shows mapping rates of WGBS samples by the three mappers; ESC=Embryonic stem cell, iPSC=induced pluripotent stem cell, Br=brain, d=day and y=year (cf. Br-5y means 5 years old brain)

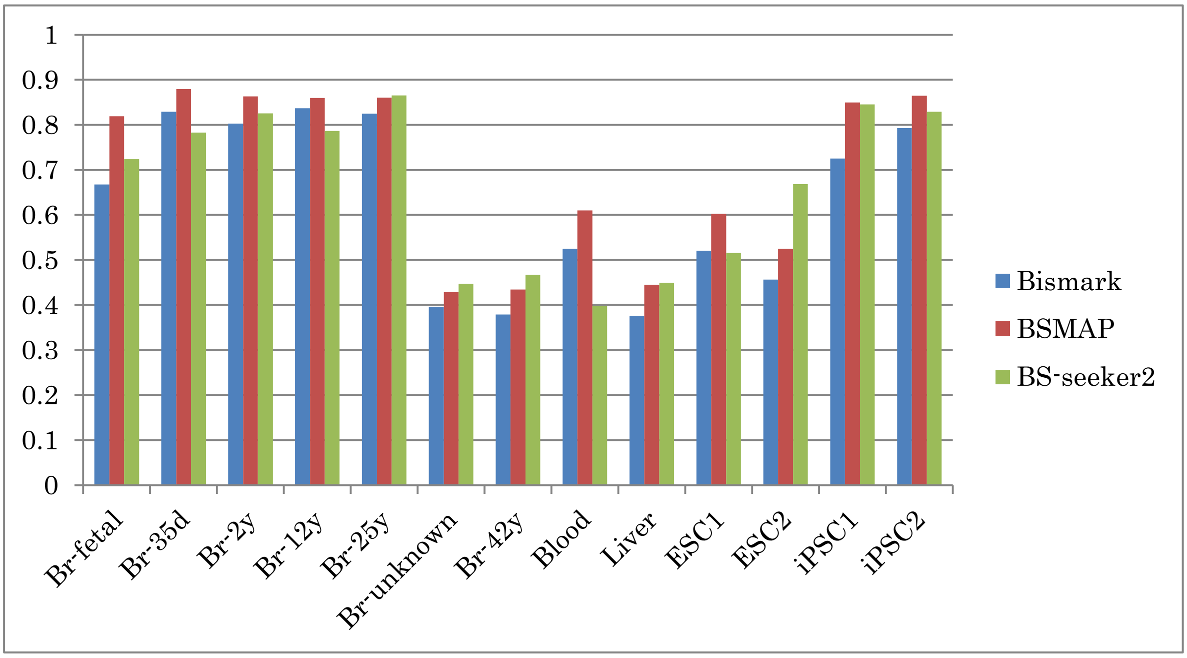

Supplement: Additional file 5 — Figure S4 - Mapping rate of whole genome bisulfite sequencing data. It shows mapping rates of WGBS samples by the three mappers; ESC = Embryonic stem cell, iPSC=induced pluripotent stem cell, Br = brain, d = day and y = year (cf. Br-5 y means 5 years old brain) (Format: PDF) [file 1471-2164-16-S12-S14-S5.pdf]
