# Supplementary material for: An integrative approach for efficient analysis of whole genome bisulfite sequencing data
Source: BMC Genomics. 2015 Dec 9;16(Suppl 12):S14. doi: 10.1186/1471-2164-16-S12-S14 (PMC4682396; doi:10.1186/1471-2164-16-S12-S14)

Additional file 6: Figure S5- Hierarchical clustering results base on CpG and CpH methylation levels extracted by BS-seeker2

Hierarchical clustering results base on CpG and CpH methylation levels extracted by BS-seeker2; Distance is 1 − spearman correlation coefficient. ESC=Embryonic stem cell, iPSC=induced pluripotent stem cell, Br=brain, d=day and y=year (cf. Br-5y means 5 years old brain). Also, the red circle groups the two samples that produced by same experiment.

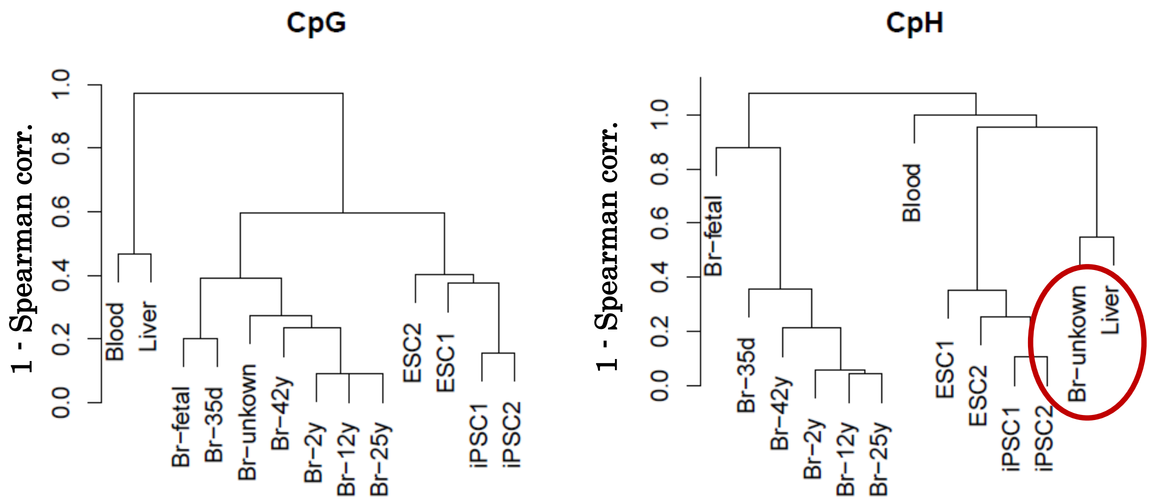

Supplement: Additional file 6 — Figure S5 - Hierarchical clustering results base on CpG and CpH methylation levels extracted by BS-seeker2. Hierarchical clustering results base on CpG and CpH methylation levels extracted by BS-seeker2; Distance is 1-spearman correlation coefficient. ESC = Embryonic stem cell, iPSC = induced pluripotent stem cell, Br = brain, d = day and y = year (cf. Br-5 y means 5 years old brain). Also, the red circle groups the two samples that produced by same experiment. (Format: PDF) [file 1471-2164-16-S12-S14-S6.pdf]
