# Supplementary material for: An integrative approach for efficient analysis of whole genome bisulfite sequencing data
Source: BMC Genomics. 2015 Dec 9;16(Suppl 12):S14. doi: 10.1186/1471-2164-16-S12-S14 (PMC4682396; doi:10.1186/1471-2164-16-S12-S14)

Additional file 7: Figure S6 – Correlation of CpG methylation levels among brain samples that produced from same experiment and different experiments

Spearman correlation of CpG methylation in gene-body regions between brain samples that produced from same experiment (GSE47966, 5 samples) and multiple experiments (GSE47966, GSE46710, GSE46698, 7 samples). Error bars represent maximum and minimum correlation value between samples. The information of gene-body regions was downloaded from refseq database.

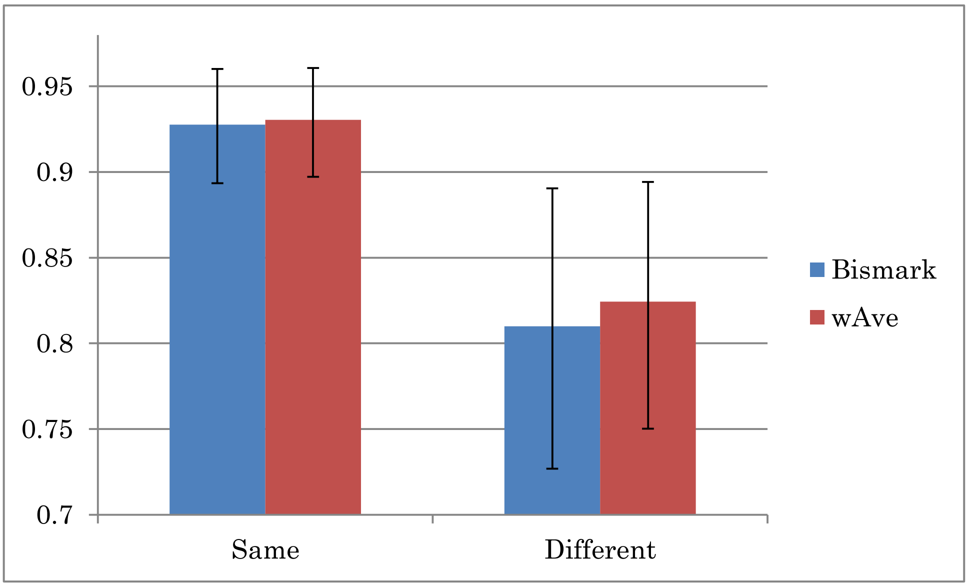

Supplement: Additional file 7 — Figure S6 - Correlation of CpG methylation levels among brain samples that produced from same experiment and different experiments. Spearman correlation of CpG methylation in gene-body regions between brain samples that produced from same experiment (GSE47966[6], 5 samples) and multiple experiments (GSE47966[6], GSE46710[28], GSE46698[29], 7 samples). Error bars represent maximum and minimum correlation value between samples. The information of gene-body regions was downloaded from refseq database. (Format: PDF) [file 1471-2164-16-S12-S14-S7.pdf]
